# Tailored therapy for *Helicobacter pylori* eradication: A systematic review and meta-analysis

**DOI:** 10.3389/fphar.2022.908202

**Published:** 2022-09-08

**Authors:** Qin Ma, Hancong Li, Jing Liao, Zhaolun Cai, Bo Zhang

**Affiliations:** ^1^ Division of Gastrointestinal Surgery, Department of General Surgery, West China Hospital, Sichuan University, Chengdu, China; ^2^ West China School of Medicine, West China Hospital, Sichuan University, Chengdu, China; ^3^ West China School of Nursing, Sichuan University, Chengdu, China; ^4^ Gastric Cancer Center, West China Hospital, Sichuan University, Chengdu, China

**Keywords:** eradication, *Helicobacter pylori*, *H. pylori*, microbial sensitivity tests, personalized therapy, tailored therapy, susceptibility-guided treatment

## Abstract

**Background:** Due to an increase in drug resistance, the eradication rate of *H. pylori* with empirical therapy has declined. Tailored therapy has been proposed as an alternative to standard empirical treatments. The necessity of personalized eradication therapy remains unclear. The aim of this study was to determine whether tailored therapy is superior to empirical therapy for *H. pylori* infection.

**Methods:** We searched for eligible randomized controlled trials in the PubMed, Embase (Ovid), Wanfang, and Cochrane Central Register of Controlled Trials databases up to 10 December 2021. A random effects model comparing pooled relative risks (RRs) with 95% confidence intervals (CIs) was applied in the meta-analysis.

**Results:** Twenty-one studies were included in the meta-analysis. In the first-line treatment, tailored therapy was more effective than empirical therapy (RR, 1.14 [95% CI, 1.08–1.21], I^2^ = 72.2%). In the second-line therapy setting, the results did not reveal significant differences between the two treatments (RR, 1.05 [95% CI, 0.84–1.30], I^2^ = 80.6%). A similar result was observed in mixed second- and third-line treatments (RR, 1.03 [95% CI, 0.96–1.11], I^2^ = 0.0%). Regarding adverse events, no significant differences were found between the two treatments (RR, 0.90 [95% CI, 0.80–1.01], I^2^ = 35.7%). Most of the results were highly heterogeneous.

**Conclusion:** A tailored approach might provide a better eradication rate than empirical methods in first-line treatment. There might be no obvious advantage in second-line or mixed second- and third-line treatments third-line treatment. Due to the high heterogeneity, the results should be interpreted with caution. Further clinical studies are needed and justified.

## 1 Introduction

*Helicobacter pylori* (*H. pylori*) is a major pathogenic factor for chronic gastritis, duodenal ulcer, gastric mucosa-associated lymphoid tissue lymphoma, gastric cancer, and other types of gastric and extragastric diseases (Fischbach and Malfertheiner, 2018). Since its discovery in 1982, the treatment of *H. pylori* infection has continued to evolve and remains a global research topic (O’connor et al., 2017). Globally, triple therapy containing pump proton inhibitors (PPIs), amoxicillin, and clarithromycin used to be the most frequently recommended primary eradication therapy for *H. pylori*. However, due to an increase in antibiotic resistance, the efficacy of triple therapy has drastically declined (Mégraud, 2004; Graham and Shiotani, 2008; Li et al., 2015; Lee and Park, 2016; Yeo et al., 2018). Thus, tailored susceptibility-guided treatment has been suggested to overcome antibiotic resistance (Fallone et al., 2016; Malfertheiner et al., 2017; Liou et al., 2018a). In tailored therapy, suitable medications are selected according to the results of susceptibility testing to achieve better eradication efficacy. Currently, the terms “tailored therapy,” “susceptibility-guided treatment,” “personalized eradication therapy,” and “customized *H. pylori* therapy” are often used interchangeably. Additionally, bismuth quadruple therapy is gradually being recommended as a first-line treatment (the initial therapy) (Fallone et al., 2016; Chey et al., 2017; Malfertheiner et al., 2017; Nyssen et al., 2021).

Antibiotic sensitivity was mainly detected by the following two methods: one was phenotypic identification, that is, *H. pylori* was cultured by gastroscopic biopsy, and antibiotic sensitivity was detected by agar dilution, disk diffusion, or E-test. The agar dilution method is the gold standard, but it is time-consuming and laborious (Ogata et al., 2014). The disk diffusion method and E-test are easy to apply, but the disk diffusion method may be difficult to explain (Lang and García, 2004; Miftahussurur et al., 2020; Tang et al., 2020). Moreover, the Clinical and Laboratory Standards Institute (CLSI) does not prescribe a standardized break point for antibiotics except for clarithromycin (Gingold-Belfer et al., 2021). On the other hand, the European Committee for Antimicrobial Sensitivity Testing (EUCAST) recommends an E-test and provides minimum inhibitory concentration (MIC) thresholds for six antibiotics (Alarcón et al., 2017). Another method is genotyping, by molecular detection (real-time PCR and fluorescence *in situ* hybridization) from stool and stomach biopsy specimens. Point mutations associated with resistance to specific antibiotics are usually detected through kits (Gingold-Belfer et al., 2021). In addition, high-throughput whole-genome sequencing techniques have been used to identify drug-resistant mutations (Chen et al., 2018; Nezami et al., 2019).

Despite the commercial availability and guideline recommendations to perform a susceptibility test, data to support this practice are scarce (Fallone et al., 2016; Chey et al., 2017; Malfertheiner et al., 2017). At present, there are three meta-analyses evaluating the effects of tailored therapy versus empirical therapy for *H. pylori* eradication (Lopez-Gongora et al., 2015; Chen et al., 2016; Gingold-Belfer et al., 2021). Lopez-Gongora et al. (2015) published the first meta-analysis in June 2015, which included 12 randomized controlled trials (RCTs) and quasi-RCTs published before February 2015, and their results showed that the evidence supporting the widespread use of customized *H. pylori* therapy, either as a first-line treatment or as a remedial treatment, was too limited. Another meta-analysis published by Chen et al. (2016) included 13 RCTs and controlled clinical trials (CCTs) published before October 2015, and their conclusions support tailored therapy as a better alternative to *H pylori* eradication. The latest study, a meta-analysis of 16 RCTs published before May 2020 by Boltin et al., found that tailored therapy was slightly better than empirical first-line triple therapy but not better than empirical first-line quadruple therapy or empirical rescue therapy (Gingold-Belfer et al., 2021). However, this study missed some studies published in the past 5 years.

Thus, we conducted a systematic review and meta-analysis without language restriction to systematically review RCTs (excluding quasi-RCTs and CCTs) comparing tailored *H. pylori* eradication regimens with empirical therapies and evaluate the effects of the two approaches for *H. pylori* eradication.

## 2 Materials and methods

This meta-analysis was conducted in accordance with the PRISMA 2020 guidelines (Supplementary Table S1) (Page et al., 2021) and the standard methodology recommended by the Cochrane Collaboration (Cumpston et al., 2019; Higgins et al., 2019). The protocol of this meta-analysis has been registered at the International Platform of Registered Systematic Review and Meta-analysis Protocols (INPLASY) (Registration number: INPLASY202230166).

### 2.1 Inclusion and exclusion criteria

This meta-analysis included only published RCTs comparing the eradication effectiveness of tailored and empirical regimens. The empirical group received standard triple therapy or other guideline recommended empirical therapies (such as sequential therapy or bismuth quadruple therapy), while the tailored group received individualized treatment through a drug sensitivity test. The recorded primary endpoint of eradication success was measured at least 4 weeks following treatment. We excluded articles unrelated to the topic of *H. pylori* eradication, conference articles, comparisons between empirical therapies, and studies with overlapping datasets. We also excluded quasi-RCTs, wherein the method for allocating participants to different interventions was not strictly random (e.g., by date of birth, day of the week, month of the year, medical record number, or order of inclusion in the study) (Cai et al., 2022).

### 2.2 Search strategy

We searched the PubMed, Embase Ovid, Wanfang, and the Cochrane Central Register of Controlled Trials (CENTRAL) databases for relevant RCTs published from their inception to 10 December 2021. The search terms were mainly as follows: tailored therapy, susceptibility-guided treatment, resistance-guided therapy, *Helicobacter pylori* eradication, and randomized controlled trial. The search strategy is detailed in Supplementary Table S2. We also searched ClinicalTrials.gov (https://clinicaltrials.gov/) for completed and ongoing trials. In addition, we searched the reference lists of included trials and review articles to identify additional studies meeting the eligibility criteria (Cai et al., 2018).

### 2.3 Study selection process

Two reviewers identified and reviewed full-text articles that were deemed relevant by screening the list of titles and abstracts. Disagreements were resolved by a team discussion.

### 2.4 Data extraction

Data extraction was conducted by two reviewers independently with a standardized form. The third author acted as a supervisor. When multiple studies were conducted on the same subjects, only the study with the highest methodological quality, the most complete results, or the most recent published date were included (Cai et al., 2021).

### 2.5 Risk of bias assessment

Two reviewers assessed the risk of bias of the included studies with the Cochrane Collaboration’s Risk of Bias assessment tool (Higgins et al., 2011) independently and resolved disagreements by discussion. Studies were assessed from the following six methodological aspects: random sequence generation and allocation concealment (selection bias), blinding of participants and personnel (performance bias), blinding of outcome assessment (detection bias), incomplete outcome data (attrition bias), selective reporting (reporting bias), and other biases.

### 2.6 Assessment of reporting biases

We created funnel plots to assess the reporting bias for the outcomes with more than 10 studies in our meta-analysis and examined this for asymmetry according to the *Cochrane Handbook for Systematic Reviews of Interventions* (Higgins et al., 2019). We used Egger’s test to determine the statistical significance of funnel plot asymmetry (Higgins et al., 2019). *p* < 0.05 suggests the presence of publication bias.

### 2.7 Statistical analysis

The primary outcome of our research was efficacy by intention-to-treat (ITT) analysis. The secondary outcome was efficacy by per protocol (PP) analysis. The safety outcome was adverse events (AEs). The meta-analyses were performed in STATA version 15.0 software (STATA, College Station, TX). We analyzed dichotomous data using risk ratios. Statistical significance was defined as *p* < 0.05. We used a random effects model by default because a certain degree of clinical heterogeneity (variability in the participants and interventions) is expected among studies. The studies were not all estimating the same intervention effect, and such intervention effects follow a normal distribution across studies (Cai et al., 2022). The outcomes with more than 10 studies were explored by subgroup analyses. In addition, the characteristics of the included studies were analyzed.

A chi-square-based Q-test was used to check heterogeneity. The I^2^ test was used to quantify the effect of heterogeneity. For chi-squared values with *p* < 0.1, heterogeneity was considered to be significantly high. The I^2^ value of 0% to 40% represents not important, 30% to 60% moderate, and 50% to 90% substantial heterogeneity (Cumpston et al., 2019).

## 3 Results

### 3.1 Study selection

**FIGURE 1:**
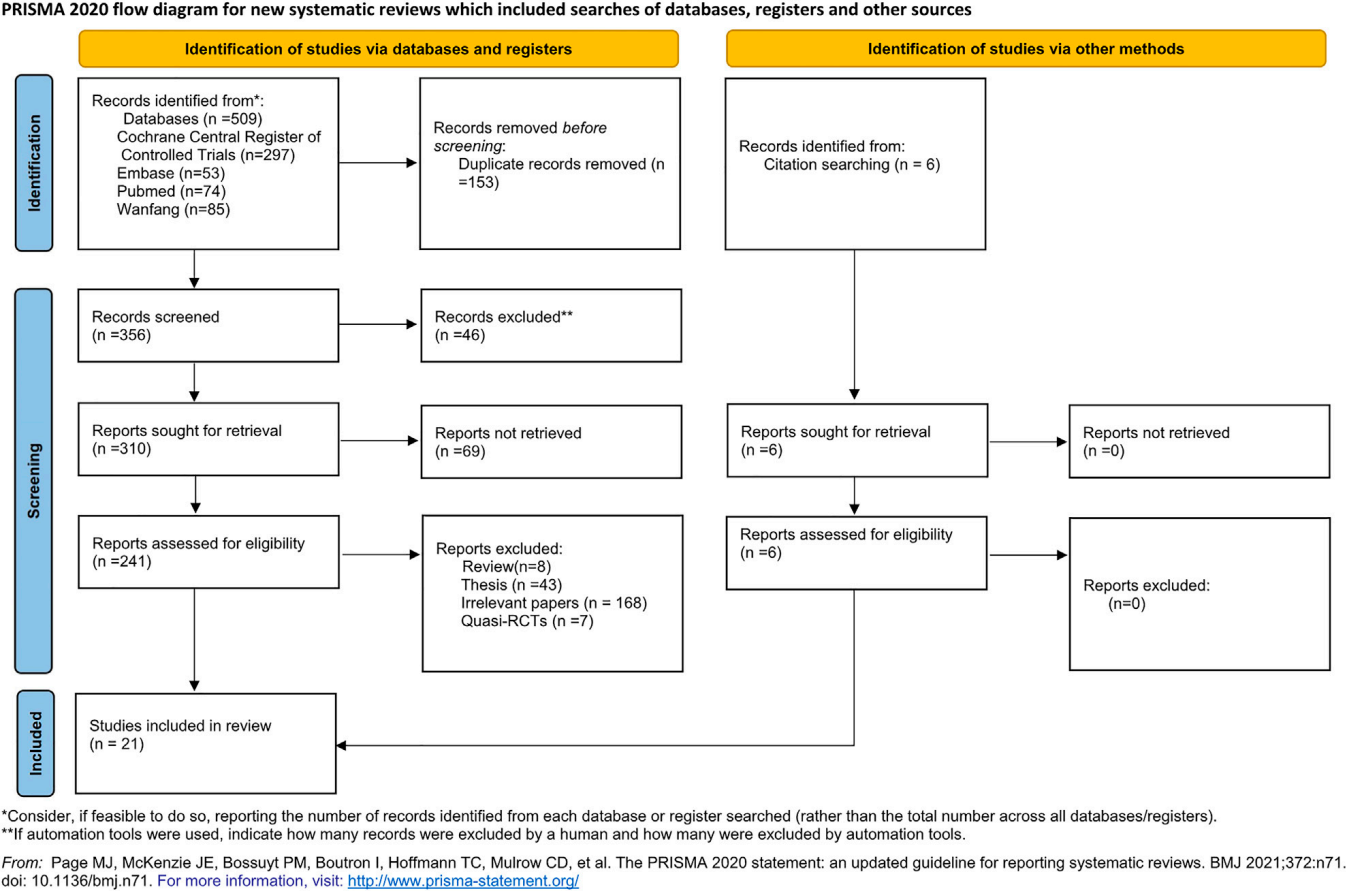
Study flow diagram.

A total of 509 titles and abstracts were identified by the screening electronic search strategy, 153 of which were duplicates. A total of 334 articles were excluded after screening the abstract and partial full text. Twenty-two full-text articles met the eligibility for assessment. In addition, we manually retrieved six citation studies that met the requirements. Finally, after reading the full text, seven quasi-RCTs were excluded. Twenty-one articles describing 22 RCTs met the inclusion criteria and were included in the quantitative synthesis meta-analysis (Avidan et al., 2001; Lamouliatte et al., 2003; Miwa et al., 2003; Neri et al., 2003; Romano et al., 2003; Marzio et al., 2006; Furuta et al., 2007; Bontems et al., 2011; Park et al., 2014; Dong et al., 2015; Zhang et al., 2015; Zhou et al., 2016; Liou et al., 2018b; Chen et al., 2019; Fan et al., 2019; Ong et al., 2019; Delchier et al., 2020; Ji et al., 2020; Kim et al., 2020; Pan et al., 2020; Perkovic et al., 2021). Figure 1 details the study selection procedure in a PRISMA 2020 flow diagram (Haddaway and McGuinness).

### 3.2 Characteristics of the included studies

Overall, 3033 participants were enrolled in susceptibility-guided treatment, and 3131 participants were enrolled in empirical treatment. In the ITT analysis of first-line therapy, the overall eradication rate was 84.8% (1898/2239) in the tailored therapy group and 74.7% (1701/2277) in the empirical group. In the PP analysis, it was 89.7% (1867/2081) vs. 80.3% (1666/2074) in the tailored and empirical groups, respectively. Additionally, in the ITT efficacy analysis of the second-line treatment (the therapy tried when the first-line therapy does not work adequately), the eradication rate was 80.2% (219/273) in the tailored therapy group and 67.1% (230/343) in the control group. The results of the PP analysis were 83.6% (199/238) vs. 71.6% (219/306) in the tailored and empirical groups, respectively. Moreover, in the second- or third-line treatment (the therapy beyond second-line therapy), the outcome was 79.1% (345/436) in the tailored group and 76.6% (333/435) in the empirical group. However, this set of results in the PP analysis was 85.4% (311/364) and 87.7% (329/375). In terms of the treatment cohort, only one study (4.8%) was conducted in children under 18 years of age (Bontems et al., 2011), and the remaining studies were conducted in adults. Regarding the treatment regions, 14 studies (66.7%) reported treatment in Asia, and the remaining studies reported treatment in Europe. Susceptibility testing was performed with bacterial culture in 18 studies (85.6%) and with molecular methods in three studies (14.3%). Patients were treatment-naive in 15 trials (68.2%), and treatment was experienced in seven trials (31.8%). Of the seven studies that included previously treated subjects, four studies included subjects with one prior treatment failure and three trials included subjects with at least one prior treatment failure. An additional two trials (9.1%) included both naive and experienced subjects. The characteristics of the included RCTs are outlined in Table 1. The included studies showed significant heterogeneity in baseline characteristics due to discrepancies in diagnostic methods, treatment regimens, geography, drug resistance, and number of previous treatment attempts of participants.

**TABLE 1:** Characteristics of studies included.

Author	Year	Susceptibility-guided treatment success n/N (%)		Empirical treatment success n/N (%)		Region	Study design	Tailored determinant	Method for determining antibiotic susceptibility	ITT sample size (tailored/empiric)
		ITT	PP	ITT	PP					
First-line treatment										
Bontems *p*	2011	71.9% (59/82)	80.8% (59/73)	81.9% (68/83)	88.3% (68/77)	Belgium	RCT	Susceptibility test	E-test	165 (82/83)
						France				
						Italy				
Chen Q	2019	91.6% (262/286)	97.7% (250/256)	85.4% (82/96)	97.6% (81/83)	China	RCT	Susceptibility test	Agar dilution method	382 (286/96)
Delchier JC	2020	85.5% (177/207)	86.5% (173/200)	73.1% (152/208)	74.4% (151/203)	France	RCT	Susceptibility test	GenoType HelicoDR	526 (266/260)
Dong F	2015	91.1% (41/45)	95.3% (41/43)	73.3% (33/45)	78.6% (33/42)	China	RCT	Susceptibility test	E-test + PCR	90 (45/45)
Furuta T	2007	96.0% (144/150)	96.6% (144/149)	70.0% (105/150)	72.9% (105/144)	Japan	RCT	Susceptibility test	PCR	300 (150/150)
Kim JL	2020	88.9% (32/36)	97.0% (32/33)	75.0% (27/36)	81.8% (27/33)	South Korea	RCT	Susceptibility test	E-test or agar dilution method	72 (36/36)
Ong S	2019	81.6% (164/201)	86.5% (154/178)	86.2% (169/196)	90.2% (157/174)	South Korea	RCT	Susceptibility test	Agar dilution method	423 (211/212)
Pan J	2020	76.8% (238/310)	83.2% (238/286)	63.7% (100/157)	68.5% (100/146)	China	RCT	Susceptibility test	Agar dilution method	467 (310/157)
Park CS	2014	94.7% (54/57)	96.4% (54/56)	71.9% (41/57)	73.2% (41/56)	South Korea	RCT	Susceptibility test	Agar dilution method	114 (57/57)
Perkovic N	2021	92.5% (37/40)	100.0% (36/36)	70.0% (28/40)	87.5% (28/32)	Croatia	RCT	Susceptibility test	E-test	80 (40/40)
Zhou L	2016	88.7% (282/318)	93.3% (278/298)	77.9% (545/700)	87.2% (524/601)	China	RCT	CYP2C19 + susceptibility test	E-test	1080 (318/700)
Fan X	2019	77.8% (210/270)	86.4% (210/243)	65.3% (179/274)	70.2% (179/255)	China	RCT	Susceptibility test	PCR + sequencing method	551(277/274)
Marzio L	2006	95.1% (39/41)	95.1% (39/41)	92.4% (36/39)	92.4% (36/39)	Italy	RCT	Susceptibility test	Agar dilution method	80 (41/39)
Neri M	2003	72.7% (88/121)	75.9% (88/116)	64.5% (78/121)	67.2% (78/116)	Italy	RCT	Susceptibility test	E-test	242 (121/121)
Romano M	2003	94.6% (71/75)	97.3% (71/73)	77.3% (58/75)	79.4% (58/73)	Italy	RCT	Susceptibility test	E-test	150 (75/75)
Second-line treatment										
Miwa H	2003	81.6% (31/38)	83.3% (30/36)	92.4% (36/39)	94.7% (36/38)	Japan	RCT	CYP2C19 + susceptibility test	Dry plate method	82 (41/41)
Zhang L	2015	75.8% (47/62)	79.7% (47/59)	84.1% (53/63)	88.3% (53/60)	China	RCT	CYP2C19 + susceptibility test	E-test	135 (67/68)
Lamouliatte H	2003	74.3% (84/113)	78.3% (65/83)	48.3% (83/172)	51.8% (72/139)	France	RCT	Susceptibility test	E-test	287 (114/173)
Avidan B	2001	80.0% (4/5)	80%.0% (4/5)	100.0% (5/5)	100%.0% (5/5)	Israel	RCT	Susceptibility test	E-test	10 (5/5)
Marzio L	2006	98.0% (50/51)	98.0% (50/51)	81.3% (26/32)	81.3% (26/32)	Italy	RCT	Susceptibility test	Agar dilution method	83 (51/32)
Furuta T	2007	75.0% (3/4)	75.0% (3/4)	84.4% (27/32)	84.4% (27/32)	Japan	RCT	Susceptibility test	PCR	36 (4/32)
Second- or third-line treatment										
Liou JM A	2018	81.0% (17/21)	88.9% (16/18)	75.0% (15/20)	78.9% (15/19)	Taiwan	RCT	Susceptibility test	Agar dilution method + PCR	41 (21/20)
Liou JM B	2018	80.0% (164/205)	83.8% (160/191)	79.0% (162/205)	87.8% (158/180)	Taiwan	RCT	Susceptibility test	Agar dilution method + PCR	510 (205/205)
Ji CR	2020	78.10% (164/210)	87.10% (135/155)	74.29% (156/210)	88.64% (156/176)	China	RCT	Susceptibility test	Agar dilution method	420 (210/210)

Abbreviation: ITT, intention-to-treat; PP, per-protocol; RCT, randomized controlled trial; PCR, polymerase chain reaction.

### 3.3 First-line treatment efficacy

**FIGURE 2:**
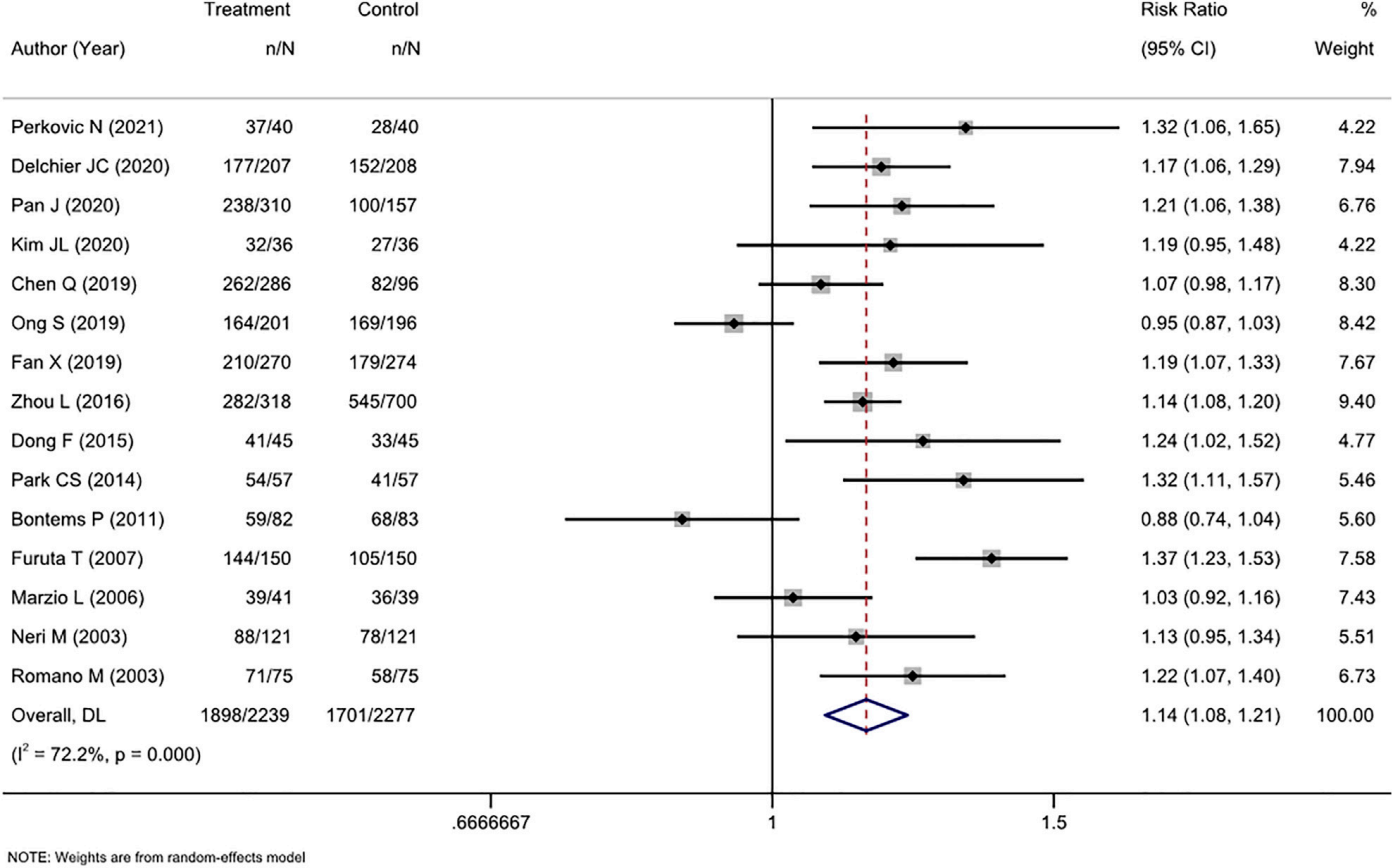
Forest plot of the ITT efficacy of RCTs comparing tailored treatment with empirical treatment in the first-line treatment.

ITT analysis of 15 RCTs (4516 patients) showed that cure rates in tailored therapy were superior to those of empirical treatment (RR, 1.14 [95% CI, 1.08–1.21], I^2^ = 72.2%; Figure 2). PP analysis also showed that the cure rates were significantly high in the tailored group (RR, 1.13 [95% CI, 1.06–1.19], I^2^ = 80.5%; Supplementary Figure S1).

The level of heterogeneity in both analyses was substantial (I^2^ 72.2%–80.5%). The *p*-values in Egger’s test for the ITT and PP analyses were 0.443 and 0.026, respectively. Combined with the funnel plots (Supplementary Figures S4, S5), it was found that there was publication bias in the included studies for the PP analysis of efficacy between the two groups.

We performed subgroup analyses in terms of the antibiotic resistance detection method, the reported region, and the type of experiential treatment received by the investigator. Regarding the detection method, both molecular and culture methods were more efficacious than empirical therapy. Among studies in which the control group received triple therapy, susceptibility-guided therapy was more effective than empirical triple therapy (RR, 1.21 [95% CI, 1.10–1.32], I^2^ = 64.2%) but not more effective than non-bismuth quadruple therapy (RR, 1.01 [95% CI, 0.84–1.22], I^2^ = 78.1%). Additionally, the tailored therapy also showed an advantage in efficiency compared to bismuth quadruple therapy (RR, 1.14 [95% CI, 1.10–1.19], I^2^ = 0.0%). This result was also confirmed by the PP analysis. Regionally, the tailored therapy yielded significantly better results than empirical therapy in Asian and non-Asian areas. Similarly, this conclusion was confirmed by both ITT and PP analyses (Table 2).

**TABLE 2:** Summary of subgroup analyses.

Group	Number of studies	ITT				PP			
		Pooled estimate		Tests of heterogeneity		Pooled estimate		Tests of heterogeneity	
		RR	95% CI	I^2^ (%)	*p*-value	RR	95% CI	I^2^ (%)	*p*-value
Overall	15	**1.14**	**1.08, 1.21**	**72.2**	**0.000**	**1.13**	**1.06, 1.19**	**80.5**	**0.000**
Antibiotic resistance detection method									
Culture method	12	**1.12**	**1.05, 1.19**	**68.2**	**0.000**	**1.09**	**1.03, 1.15**	**73.5**	**0.000**
Molecular method	3	**1.24**	**1.12, 1.36**	**60.6**	**0.079**	**1.24**	**1.15, 1.33**	38.3	0.198
Empirical therapy									
Triple therapy	6	**1.21**	**1.10, 1.32**	**64.2**	**0.016**	**1.20**	**1.11, 1.30**	**58.5**	**0.034**
Bismuth quadruple therapy	5	**1.14**	**1.10, 1.19**	0.0	0.435	**1.12**	**1.03, 1.22**	**84.3**	**0.000**
Non-bismuth quadruple therapy	3	1.01	0.84, 1.22	**78.1**	**0.010**	0.99	0.89, 1.12	**65.6**	**0.055**
Other	1	1.13	0.95, 1.34	-		1.13	0.96, 1.33	-	-
Region									
Asian	9	**1.17**	**1.08, 1.26**	**77.4**	**0.000**	**1.15**	**1.06, 1.23**	**86.2**	**0.000**
Non-Asian	6	**1.11**	**1.01, 1.23**	**65.7**	**0.012**	**1.10**	**1.01, 1.19**	**60.3**	**0.028**

Abbreviation: CIs, confidence intervals; ITT, intention-to-treat; PP, per-protocol; RR, relative risk.

The bold value represents statistically significant data.

### 3.4 Non-first-line treatment efficacy

#### 3.4.1 Second-line treatment

**FIGURE 3:**
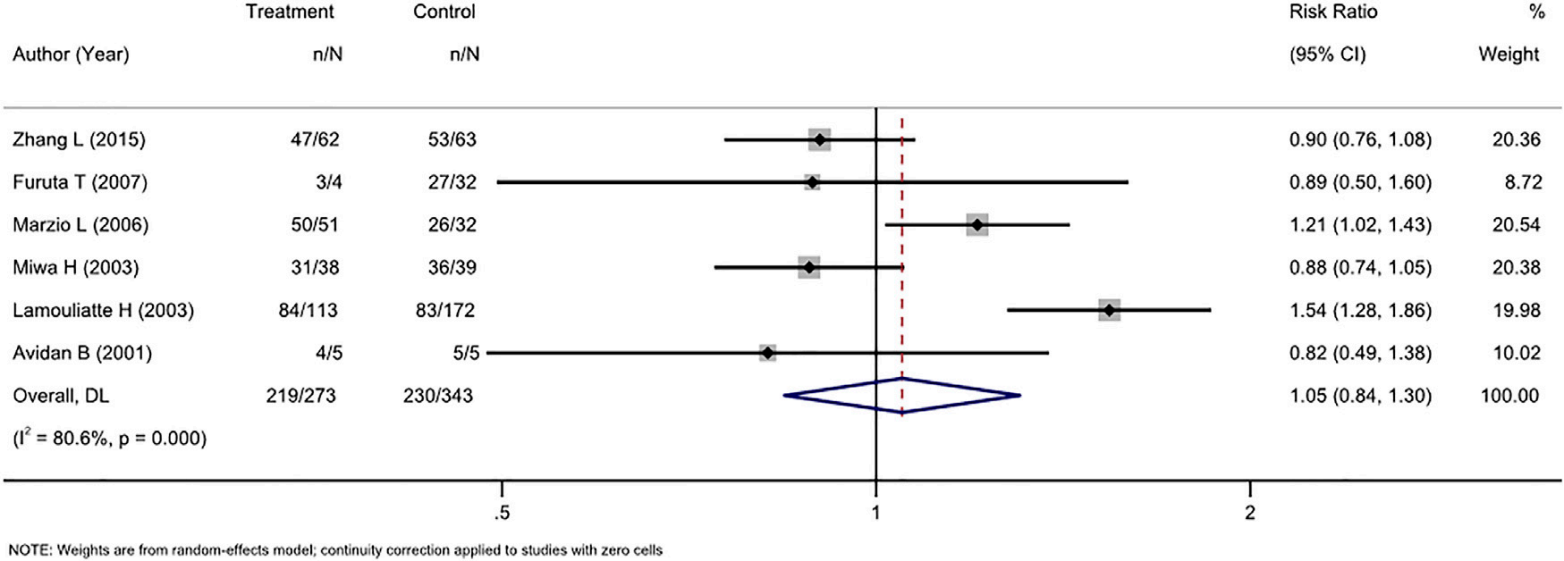
Forest plot of the ITT efficacy of RCTs comparing tailored treatment with empirical treatment in the second-line treatment.

Six RCTs compared tailored therapy with empirical treatment as the second-line treatment. ITT analysis of 616 patients did not reveal significant differences between the two therapy strategies (RR, 1.05 [95% CI, 0.84–1.30], I^2^ = 80.6%; Figure 3). PP analysis yielded similar results (RR, 1.04[95% CI, 0.85–1.29], I^2^ = 80.0; Supplementary Figure S2).

There was substantial heterogeneity (I^2^ 80%–80.6%) in the analyses; this fact, along with the limited number of patients, ruled out the use of subgroup analysis and funnel plot analysis.

#### 3.4.2 Mixed second- and third-line treatments

**FIGURE 4:**
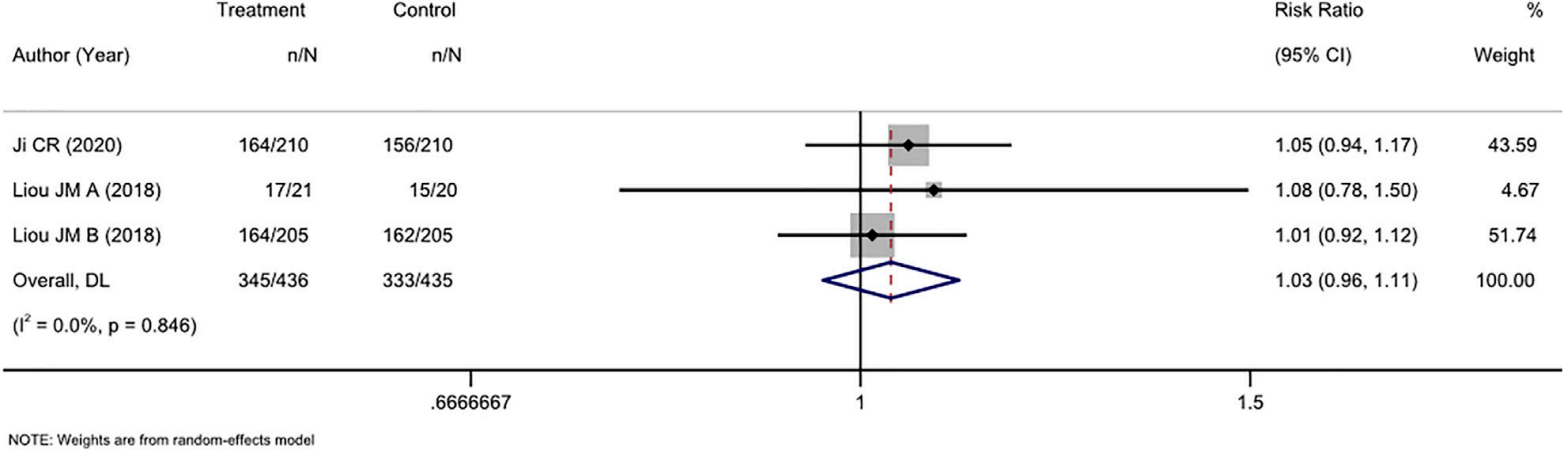
Forest plot of the ITT efficacy of RCTs comparing tailored treatment with empirical treatment in the mixed second- and third-line treatment.

Three RCTs compared tailored therapy with empirical treatment as mixed second- and third-line treatments. ITT analysis of 871 patients did not show significant differences between the two therapy strategies (RR, 1.03 [95% CI, 0.96–1.11], I^2^ = 0.0%; Figure 4). PP analysis showed similar results (RR, 0.97[95% CI, 0.92–1.03], I^2^ = 0.0%; Supplementary Figure S3).

### 3.5 Adverse events

**FIGURE 5:**
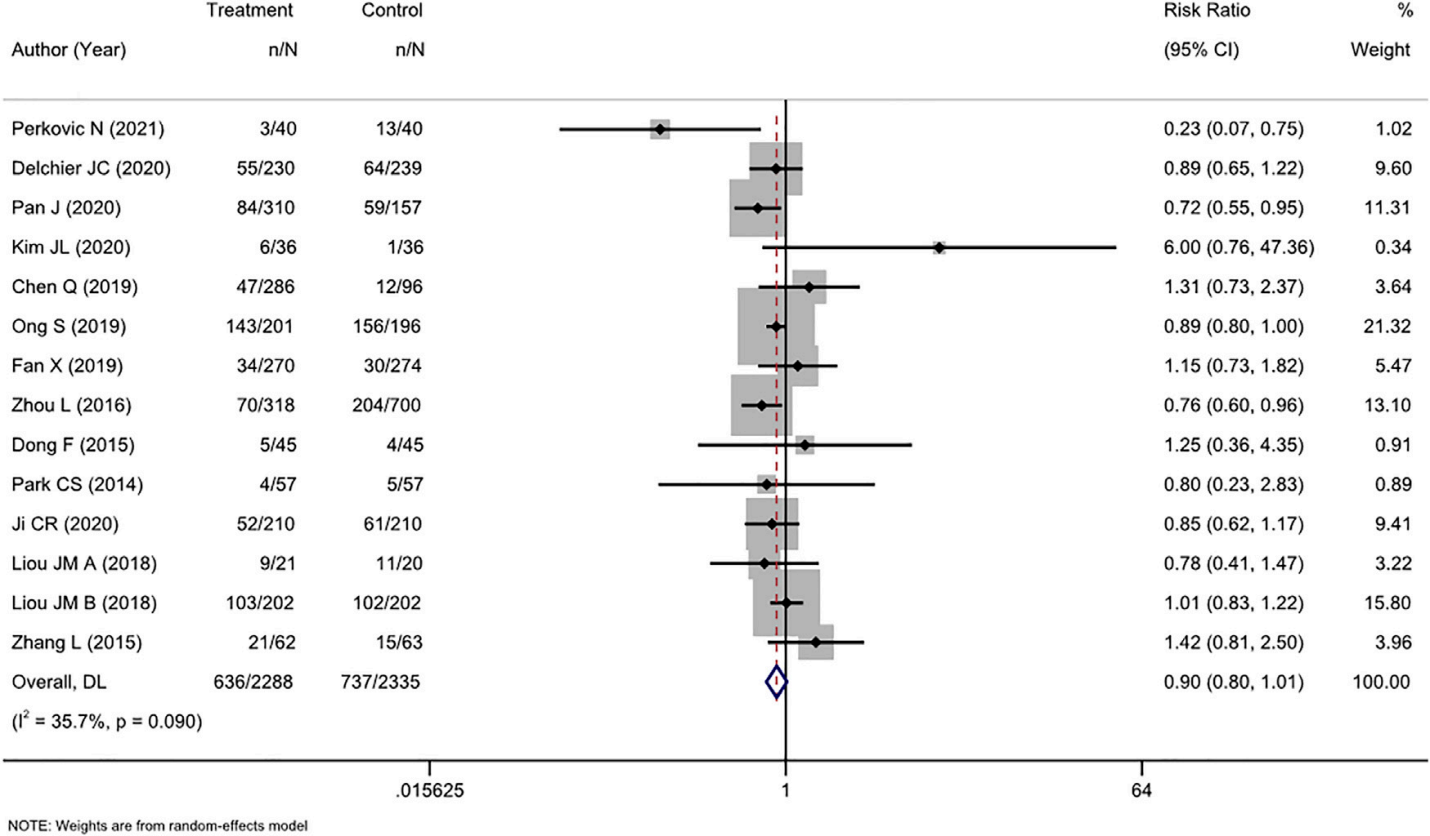
Forest plot of the adverse events of RCTs comparing tailored treatment with empirical treatment.

AEs were comprehensively reported in 16 studies, and two (Avidan et al., 2001; Furuta et al., 2007) of these studies reported no AEs. Therefore, a meta-analysis was performed on the 14 remaining studies. Overall, we found a nonsignificant trend toward fewer AEs among participants who received tailored therapy than among those who received empirical treatment (RR, 0.90 [95% CI, 0.80–1.01], I^2^ = 35.7%; Figure 5)**.** The most common AEs were taste change, nausea, vomiting, and diarrhea, which usually did not lead to treatment discontinuation.

There was moderate heterogeneity (I^2^ = 35.7%) in the analysis. The *p*-value in Egger’s test for the comparison of AEs was 0.630. Combined with the funnel plot analysis (Supplementary Figure S6), it was found that there was no publication bias in the included studies for the RR of AE rates between the two groups.

### 3.6 Risk of bias assessment

A total of 21 articles including 22 RCTs were examined in this meta-analysis. Among these, 15 had a low risk for bias in random sequence generation (selection bias, 68.2%), 9 had a low risk for bias in allocation concealment (selection bias, 40.9%), none of the studies had low risk for bias in blinding participants and personnel (performance bias, 0%), 2 had a low risk for bias in blinding the outcome assessment (detection bias, 9.1%), 21 had a low risk for bias of incomplete outcome data (attrition bias, 95.5%), and 20 had a low risk for bias of selective reporting (reporting bias, 90.9%). In general, the high risk of bias in the studies we included was chiefly in the blinding. The risk of bias was moderate to high in most of the studies and is summarized in Supplementary Table S3.

## 4 Discussion

The main result of our meta-analysis, supported by both the ITT and PP analyses, is that tailored therapies might be superior to empirical therapies in the first-line treatment. However, there were no significant differences between tailored and empirical therapies in second-line therapy and mixed second- and third-line therapy. Notably, the substantial heterogeneity in ITT and PP cure rates indicated that the results should be interpreted with caution. Because of the diversity in antibiotic resistance, diagnosis methods, and treatment regimens of *H. pylori*, the evidence included in our research was highly heterogeneous. The high heterogeneity in both first-line treatment and rescue treatments prevented us from drawing a valid conclusion.

We further conducted first-line subgroup analyses in terms of the antibiotic resistance detection method, the region, and the empirical regimen. Tailored therapies took advantage of the empirical therapies found in both the culture and molecular methods. It should be noted that, in clinical practice, it is troublesome to isolate and culture *H. pylori* successfully from gastric biopsy specimens. The results are largely dependent on the quality of clinical specimens, time interval between sampling and culture, and transportation conditions (Pohl et al., 2019). Moreover, *H. pylori* culture requires highly trained staff to take 7 days until samples can be reported as negative and 2 weeks to reveal antibiotic susceptibility results (Pohl et al., 2019). Molecular testing contrasts with traditional culture-based testing because it can use clinical isolates, fresh or formalin-fixed gastric biopsies, or stool samples and could rapidly provide data on multiple antibiotics (Graham, 2021). To effectively use these data, we need studies to identify the advantages and limitations to molecular methods using different types of samples and more comparisons between molecular- and culture-based methods concerning treatment outcomes (Graham, 2021).

Regarding regionalism, tailored treatment was significantly more effective than first-line empirical therapy in both Asian and non-Asian areas. The effect was slightly more pronounced in Asia. It is noticeable that the literature from different regions showed discrepant geographical resistance rates to antibiotics (Ierardi et al., 2013). Since the gene mutation site increasing clarithromycin resistance might differ from Asia to other continents, epidemiological data about resistance vary across regions (Oleastro et al., 2003). In China, the resistance rate ranges between 21.5% and 23.8% (Su et al., 2013). Interestingly, in Japan, the rate was at least 15%, and a much higher resistance rate was observed in another study (86.4%), suggesting that even in the same geographical region, relevant differences may occur. In Northern Europe, the trend is relatively inconspicuous (Koivisto et al., 2004; Storskrubb et al., 2006; Selgrad et al., 2013), while in Italy, the rate is not only high (24.1%) but also increases rapidly (De Francesco et al., 2007). In contrast, the percentages of amoxicillin resistance are almost negligible worldwide except in a few regions (Ierardi et al., 2013). Iran and Japan have reported resistance rates of 28.6% (Milani et al., 2012) and 8.2%–15.2% (Murakami et al., 2013), respectively. In Cameroon, the rate is specifically high (85.6%) (Murakami et al., 2013). Over the years, levofloxacin-resistant strains have increased, and an unfavorable tendency has been revealed in Asia (Ierardi et al., 2013). Resistance was detected at approximately 18.4% in Vietnam and 20.6% in China (Ierardi et al., 2013). In Europe, a multicentric epidemiologic study reported an overall percentage of 14.1% (Megraud et al., 2013), indicating that levofloxacin may be an ineffective option in the future.

In the subgroup analysis of diverse empirical methods, tailored therapy showed the best advantage over the standard triple therapy. However, current guidelines recommend the use of triple therapy as first-line treatment only in areas with clarithromycin resistance rates <15%. However, the majority of studies using triple therapy as a control group had clarithromycin resistance rates>20%. In addition, tailored therapy was superior to bismuth-quadruple therapy in terms of both ITT and PP cure rates. Nevertheless, it should be noted that in the PP analysis, the heterogeneity was high. This might be explained by the very limited evidence—only five studies were suitable for this analysis. Among them, the PP analysis of two studies from China (Chen Q et al., Zhou L et al.), both found very similar cure rates between bismuth quadruple therapy and the empirical therapy, which were inconsistent with the results of other therapies. This suggests that the relationship between the eradication rate and race can be further explored. Finally, only three studies in the control group used non-bismuth quadruple therapy, and no significant difference was found in either ITT or PP analyses. In fact, the number of studies using non-bismuth quadruple therapy or other therapies (such as sequential therapy) was too limited to conclude.

Regarding second-line or mixed second- and third-line treatments, research has been relatively limited. In total, nine RCTs met the inclusion criteria. The cure rate did not show significant differences between the two treatments. Above all, the effectiveness of tailored therapy following previous treatment failures is uncertain.

Concerning safety, we found a trend in favor of tailored therapy in terms of AEs. However, this trend was not statistically significant, which might be caused by the small sample size.

Our study has its own strengths and limitations. This meta-analysis has the following advantages. First, all the included studies were randomized controlled trials, which ensured the validity of the overall results and reduced the possibility of bias in individual studies. Second, the study selection process was rigorous, with two independent reviewers screening eligibility and reviewing to ensure the accuracy and completeness of the included literature. In addition, our data retrieval results were complete. Under strict screening conditions, our study included 22 randomized controlled trials, the largest meta-analysis of qualified studies ever conducted. Finally, we analyzed the side effects to further investigate the feasibility of tailored regimens.

Additionally, our study was also limited in that we did not conduct a health economic analysis of the two treatments to study the difference in economic cost between tailored and empirical treatments. Although two trials have shown that the tailored treatment saves more money than the standard triple therapy (saving an average of $5 and $12, respectively) (Chen et al., 2016), there are still not enough data to determine whether the custom treatment saves more money than other popular empirical regimens. In addition, we attempted to conduct a subgroup analysis of the studies with first-line treatment in terms of the clarithromycin resistance rate, but the clarithromycin resistance rate reported in most studies was > 20%, thereby preventing us from conducting the analysis. Furthermore, we did not search the Web of Science and Scopus databases, which might have caused us to miss the potentially qualified literature. Finally, the high heterogeneity in the current study prevented us from drawing a valid conclusion.

## 5 Conclusion

In conclusion, in terms of the eradication rate, tailored therapy might be a better choice than empirical therapy in first-line treatment. The safety profiles of tailored therapy and empirical treatment might be comparable. However, evidence is too limited to support the generalized use of tailored therapy for *H. pylori* treatment, either as first-line or as rescue treatment; more studies are needed to reach an evidence-based conclusion.

## Data Availability

The original contributions presented in the study are included in the article/Supplementary Material; further inquiries can be directed to the corresponding authors.
